# LncRNA H19 overexpression induces bortezomib resistance in multiple myeloma by targeting MCL-1 via miR-29b-3p

**DOI:** 10.1038/s41419-018-1219-0

**Published:** 2019-02-06

**Authors:** Yafang Pan, Yu Zhang, Wenwen Liu, Yan Huang, Xianjuan Shen, Rongrong Jing, Jiang Pu, Xudong Wang, Shaoqing Ju, Hui Cong, Hongmei Chen

**Affiliations:** 1 Department of Laboratory Medicine, Affiliated hospital of Nantong University, Nantong, Jiangsu 226001 China; 2 Department of General Surgery, Affiliated Hospital of Nantong University, Nantong, Jiangsu 226001 China; 3 Vip ward, Affiliated Hospital of Nantong University, Nantong, Jiangsu 226001 China

## Abstract

Radiotherapy, chemotherapy, autologous/allogeneic stem cell transplantation, and targeted drug therapy are currently available therapeutic options for multiple myeloma (MM), but the clinical outcome remains unsatisfactory owing to frequent occurrence of drug resistance. Anti apoptosis is one of the main mechanisms to mediate drug resistance. Studies have shown that MCL-1 plays a key role in the growth of cancer cells “escaping” drug attacks, but the underlying mechanism remains unclear. Our previous study demonstrated that lncRNA H19 was highly expressed in the serum of MM patients. Bioinformatics predicts that miR-29b-3p is the downstream target gene, and MCL-1 is the downstream target protein of miR-29b-3p. Therefore, we speculated that MCL-1 may be involved in the occurrence of drug resistance through epigenetics. On the basis of these previous findings, the present study was intended to explore the biological function of H19, interactions between the downstream target genes, and the effect of H19 on BTZ resistance of myeloma cells. In addition, in vivo experiments we have also confirmed that H19 promoted tumor growth and may develop resistance to bortezomib partly. It was found that H19 reduced cell sensitivity to the chemotherapeutic drug BTZ by working as a miRNA sponge to inhibit the expression of miR-29b-3p, enhance MCL-1 transcriptional translation and inhibit apoptosis. These findings may help gain insights into the molecular mechanism of acquired BTZ resistance and develop new drug targets for the clinical treatment of MM.

## Introduction

Multiple myeloma (MM) is a malignant disease characterized by gathering of a large number of malignant plasma cells in the bone marrow and the presence of monoclonal protein (M protein) in blood, urine, or both^1^. Chemotherapy, autologous/allogeneic stem cell transplantation, and targeted drug therapy are currently available therapeutic options for the treatment of MM patients, with the aim to improve their quality of life and prolong the survival time^2^. However, the clinical outcome remains unsatisfactory because of acquired drug resistance, which has become one of the biggest challenges in the clinical treatment of MM. Therefore, further studies are warranted to explore the molecular mechanism of acquired drug resistance in MM for the sake of developing effective coping strategies for the treatment of MM.

Bortezomib (BTZ) is a representative small-molecule proteasome inhibitor and immunomodulatory agent commonly used in the past decade to improve the remission rate, increased ease depth, and prolong the survival of MM patients^3^. However, common occurrence of primary or acquired drug resistance to BTZ has become a crux in improving the prognosis of MM patients^4^, but little progress has been made in this respect owing to the complex mechanism underlying acquired resistance to BTZ in MM. Previous studies on drug resistance are mainly concerned with the following six aspects: PSMB5 gene mutation^5^, high expression of nuclear factor (NF)-κB^6^, maturation and abnormal expression of proteasome^7^, inhibition of UPR and downregulation of XBP1 expression^8^, autophagy activation^9^, and inhibition of apopotosis^10^. With respect to PSMB5 mutation, Ri et al.^11^ found that the degree of drug resistance in transfected cell line PSMB5-tKMS-11 was lower than that in BTZ-induced KMS-11/BTZ-resistant cell lines, and the mutation was not found in some drug-resistant MM cell lines^12^ and drug-resistant MM patients^13^. Acquired BTZ resistance was also reported to be attributed to the upregulation of heat shock proteins (HSPs) such as HSP90 and HSP27, knowing that they could promote the activation of NF-κB as a ubiquitin molecular chaperone, and this expression was often found in BTZ refractory MM patients^14^. In the study of primary myeloma samples, a certain degree of NF-kappa B activity was found in all BTZ-resistant CD138^+^ patients^14^. In addition, when MM cells were co-cultured with bone marrow mesenchymal stem cells (BMSCs) from MM patients, the activity of NF-κB pathway promoting BTZ resistance was further enhanced, but it was not observed when they were co-cultured with healthy BMSCs. Although there is strong evidence that NF-kappa B plays a role in BTZ-resistant MM patients, the overall rate of missense mutations in the treated and newly diagnosed patients is not statistically significant by standard whole-genome sequencing or whole-protein coding exon sequencing^15^.

In view of the above results, inhibition of apoptosis may be more important in the process of drug resistance as compared with other drug resistance mechanisms. A recent study by Wang et al.^16^ demonstrated that miR-17-5p played a role in the development of drug resistance in gastric cancer cells, at least partially by modulating apoptosis via targeting p21. Yang et al.^17^ found that Kanglaite could reverse multidrug resistance of human hepatocellular carcinoma by inducing apoptosis and cell cycle arrest via the PI3K/AKT signaling pathway. Vazanova A et al.^18^ discovered a statistically significant increase in mRNA expression of all investigated proteins (p53, BAX, Bcl-2, and Bcl-XL) between the leukemia samples and leukocytes from healthy volunteers. It is therefore clear that some oncogenic mutations can disrupt apoptosis, leading to tumor initiation, progression or metastasis. We have good reason to believe that apoptosis is important for the occurrence of drug resistance.

Myeloid cell leukemia sequence 1 (Mcl-1) is an antiapoptotic Bcl-2 family protein with a very short half life. After ubiquitination, it is degraded by proteasome^19^. It is highly expressed in many kinds of tumors, and the expression of MCL-1 is the reason for resistance to various chemotherapeutic drugs^20^. There is evidence that MCL-1 is an important target for tumor diagnosis and treatment. For example, MCL-1 overexpression is one of the most common genetic variants observed in human lung and liver cancers^21^. In addition, overexpression of MCL-1 induced resistance to Bcl-2 inhibitors and some widely applied anticancer drugs including paclitaxel, vincristine, and gemcitabine. Studies also demonstrated that silencing MCL-1 could restore the sensitivity of drug-resistant cells^22^. MCL-1 is highly expressed in MM cells^23^. It is one of the main survival factors of MM cells, and is involved in the process of tumor drug resistance. However, its specific molecular mechanism of drug resistance is not clear.

Long non-coding RNAs (lncRNAs) refer to a class of RNA transcripts larger than 200 nucleotides with no function of encoded proteins^24^. With the development of high throughput sequencing technologies in recent years, more functional lncRNAs have been identified and gradually become the focus of research. Current LncRNA research related to MM is mainly focused on lncRNA OIP5-AS1^25^, MALAT1^26^, MEG3^27^, PCAT1^28^, KIAA0495^29^, UCA1,^30^ and CRNDE^21^. However, few studies have reported the association between LncRNAs and drug resistance in MM. Reports in the existing literature show that lncRNA H19 acts as an oncogene in most tumors and plays a role in the pathogenesis of breast cancer^31^, HCC,^32^ and bladder cancer^33^ through a variety of mechanisms such as heterotopic expression, endogenous RNA competition, and cell cycle intervention. However, the resistance mechanism of H19 in MM has not yet been reported.

The aim of the present study was to explore the molecular mechanism underlying drug resistance in MM on the basis of previous research. It was found that H19 acted as a miRNA sponge of miRNA-29b-3p to promote transcriptional translation of MCL-1. In addition, the H19/miR-29b-3p axis partly contributed to BTZ resistance of MM cells by targeting MCL-1.

## Methods

### Patients and samples

Serum samples were collected from MM patients who received treatment in Affiliated Hospital of Nantong University (Nantong, China) between November 2015 and August 2017. They included 50 newly diagnosed MM patients, 80 relapsed MM patients and re-hospitalized MM patients, 12 BTZ-resistant patients, and 67 healthy individuals. This study was approved by the ethics committee of the said hospital. All the patients and healthy subjects were informed of the study purpose, and provided informed consent for participation in the study. Blood samples were collected in vacuum extraction tubes containing separating glue and centrifuged at 3000× *g* for 15 min. The upper-level serum ~ 2 mL was placed in a non enzyme eppendorf tube and stored at − 80℃ for use.

### Cell lines and culture

Human MM cell lines H929, U266, and 8226 (Cell bank of the Chinese Academy of Sciences, Shanghai, China) were cultured in Rosewell Park Memorial Instittute (RPMI) medium 1640 (Invitrogen, Carlsbad, CA) containing 10% fetal bovine serum (Gibco, Grand Island, NY) and 1% penicillin–streptomycin at 37℃ in 5% CO_2_. Normal human marrow CD138 ^+^ plasmocytes were obtained by magnetic beads sorting (EasySepTM, Stem cell, Canada).

### RNA extraction and quantitative real-time PCR (qPCR)

Total RNA was extracted with 400 μl serum, using a serum extract Kit (Life Technologies, USA) according to the manufacturer’s protocol. Total RNA of MM cells was isolated using TRIzol reagent (Invitrogen) and quantified with a nanophotometer. RT-qPCR was utilized to evaluate the expression of lncRNA, miRNA, and mRNA in the serum samples or cells on the Roche LightCycler 480 (Roche, Switzerland). The amplification of the appropriate product was confirmed by melting curve analysis. The H19 primer sequences are as follows: 5′-GCGGGTCTGTTTCTTTACTTC-3′ (forward) and 5′-TTTCATGTTGTGGGTTCTGG-3′ (reverse). The sequences of the primers used for 18 S amplification were 5′-GTAACCCGTTGAACCCCATT-3′ (forward) and 5′-CCATCCAATCGGTAGTAGCG-3′ (reverse). The primer of miR-29b-3p was designed by Guangzhou Ruibo Company (Guangzhou, China). The MCL-1 primer sequences are as follows: 5′ -TTAAACAAAGAGGCTGGGATG-3′ (forward) and 5′ -ACCAGCTCCTACTCCAGCAA-3′ (reverse). The reactions were performed in triplicate. Samples with a CT > 40 were considered negative. Relative expression was calculated using the 2^−ΔΔCT^ method.

### Cell transfection

The Vector, pcDNA-H19, Sh-1156, Sh-NC, miR-29b-3p mimic, and mimic control were all designed and purchased from GenePharma (Suzhou, China). All mimics and plasmids were transfected into cells using Lipofectamine 3000 (Invitrogen). At 48-h post transfection, transfected cells were collected for next analysis.

### Cell migration assay

Cell migration assay was performed in 24-well Transwell chamber. We collected cells at 48 h after transfection. For cell counts, the concentration of centrifugally suspended cells was ~ 5 × 10^5^/mL. A total of500 μL of RPMI medium 1640 was added in 24-well plate, and the chamber was placed in the RPMI medium 1640 to moisten it. Next, the medium (RPMI medium 1640 containing 20% fetal bovine serum) was added to another 24-well plate. Then, we took out the moist chamber and put it in 24-well plate containing the medium. Cell suspension was prepared in RPMI medium 1640, and 150 μL of cell suspension was added to each chamber. They were incubated in a incubator incubated with 24 h~ 48 h. We used cotton swabs to remove excess cells and media from the upper chamber, and then inverted them. The cells at the bottom of the chamber were fixed in formaldehyde for 2 min and stained with 0.1% crystal violet for 15 min. Last, taking pictures and counting under the inverted microscope (at least four random fields), and statistical analysis. Each experiment were performed in triplicate.

### Cell proliferation assay

CCK8 assay was used to detect cell viability. Cells transfected with plasmids or mimics were seeded in 96-well plates at a density of 2–3 × 10^3^ cells/well with five repeating holes in each group. Cells were put in a 37℃ incubator and detected for the optical density value using a microplate reader at the designated time pints.

After being cleaned with pre-cooled phosphate-buffered saline (PBS), cells cycle analysis was performed 48 h after transfection as follows. Cells were harvested, fixed in 70% ice-cold ethanol at 4℃ for 2 h or a longer time, added with 0.5 μl propidium iodide to each tube, and bathed at 37℃ for 30 min. Red fluorescence was detected at 488 nm by flow cytometry, and the light scattering was detected at the same time. All analyses were conducted on a FACSCalibur BD flow cytometer. The data were collected and processed using the BD FACSuite analysis software.

### Apoptosis analysis

Cells were collected after 48-h transfection, washed with pre-cooled PBS twice, overhung in 1 × BindingBuffer at a concentration of 10^6^ cells/ml, and analyzed for apoptosis with 7-AAD and PE staining in a FACSCalibur BD flow cytometer.

### Nucleoplasm isolation technique

Nucleoplasm isolation was performed using the cytoplasmic & nuclear RNA purification kit (Norgen, Biotek, Canada) according to the manufacturer’s instructions. Finally, the RNA level was detected by RT-qPCR.

### RNA FISH

The FISH kit was purchased from Ribo Bio (Guangzhou), and the experiment was performed according to the manufacturer’s instructions and visualized with a fluorescence microscope.

### Western blot

Cells were lysed on ice with cold radioimmunoprecipitation assay lysis buffer (NCM Biotech, Suzhou, China) including protease inhibitors. The same amount of protein (50 μg) was subjected to 10% or 15% sodium dodecyl sulfate polyacrylamide gel electrophoresis and then transferred to polyvinylidene difluoride membranes (Millipore, Billerica, MA, USA) for band separation. Subsequently, the membranes were blotted with 10% skimmed milk in Tris-buffered saline with Tween-20 (TBST), followed by probing with the antibodies overnight at 4℃. After washing extensively with TBST on the following day, the membranes were incubated with horseradish peroxidase-conjugated goat anti-rabbit IgG antibody. Primary antibodies specific to MCL-1, BCL-2, Bim, Bax, Caspase3, C-Caspase3, PARP, C-PARP, and β-actin (1:1000; Cell Signaling Technology) were used. The blots were then incubated with goat anti-rabbit (Cell Signaling Technology) and visualized using enhanced chemiluminescence.

### Luciferase reporter assay

To observe interactions between H19 and miR- 29b-3p, wide type H19 and the mutant H19 were cloned into the Pezx-FR02 reporter vector. The Pezx-FR02 or Pezx-FR02-H19-MUT was co-transfected with miR-29b-3p mimic or miRNA mimic control. Forty-eight hours post transfection, Dual Luciferase Assay (Promega, Madison, USA) was used to determine the luciferase reporter activities according to the manufacturer’s instructions.

### IC50 determination

The transfected cells in the logarithmic phase of growth were seeded in five repeating holes into 96-well plates at a density of 3000 cells per well. After 24-h incubation, cells were treated with the indicated concentrations (20, 50, 100, 150, 200, and 500 nm) of BTZ for 48 h. Subsequently, 10 μl CCK8 solution was added to each well, followed by incubation for 1–2 h at 37℃ in 5% CO2. The absorbance at 450 nm was recorded using a microplate reader (BioTek, Winooski, VT, USA). According to the results obtained, IC50 was calculated by SPASS.

### Establishment of xenograft model in nude mice

Male athymic BALB/c mice, 5-week-old, were randomly divided into three groups. There were two nude mice in the first and second groups, and three nude mice in the third group. H929 cells with a cell concentration of 5 × 10^9^/L were inoculated subcutaneously into the left axillary of each group of nude mice at 200 μL to establish a model of subcutaneous tumor transplantation in nude mice. Nude mice were observed three times a week, and the physical condition, diet and exercise condition of nude mice were recorded. The tumor length (*a*) and width (*b*) were measured once a week from the inoculation date. The tumor volume was calculated according to the formula *V* = *a* × *b*^2^/2, and the growth curve of the tumor was drawn. After tumor formation, pcDNA-H19 and vector were injected into the first group. Bortezomib was added to the second group. K/NC, K/miRNA-29b-3p, pcDNA-H19/miRNA-29b-3p were injected into the third group respectively, which were treated with bortezomid. The injection continued 4 weeks with one time for each week. In addition, drug treatment was delayed for 1 week. One week after the last injection, the nude mice were killed and the tumor was removed. The tumor tissues were stored at − 80℃ for detecting the expression of H19 by qRT-PCR.

### Statistical analysis

All statistical analyses were performed using Graph Prism 5.0 software (GraphPad Prism, San Diego, CA). The data are shown as mean ± standard deviation. Student’s *t* test or one-way analysis of variance was used to analyze the differences between two or more groups. The results were considered statistically significant when *P* was < 0.05.

## Results

It was found in our previous study^34^ that H19 was highly expressed in MM patients and MM cells, and even more highly in the serum of BTZ-resistant patients. In response to these findings, the present study was designed to gain insights into the molecular mechanism of H19 underlying BTZ resistance in MM.

### The effect of abnormal H19 expression on the malignant biological phenotype of MM cells

**Fig. 1:**
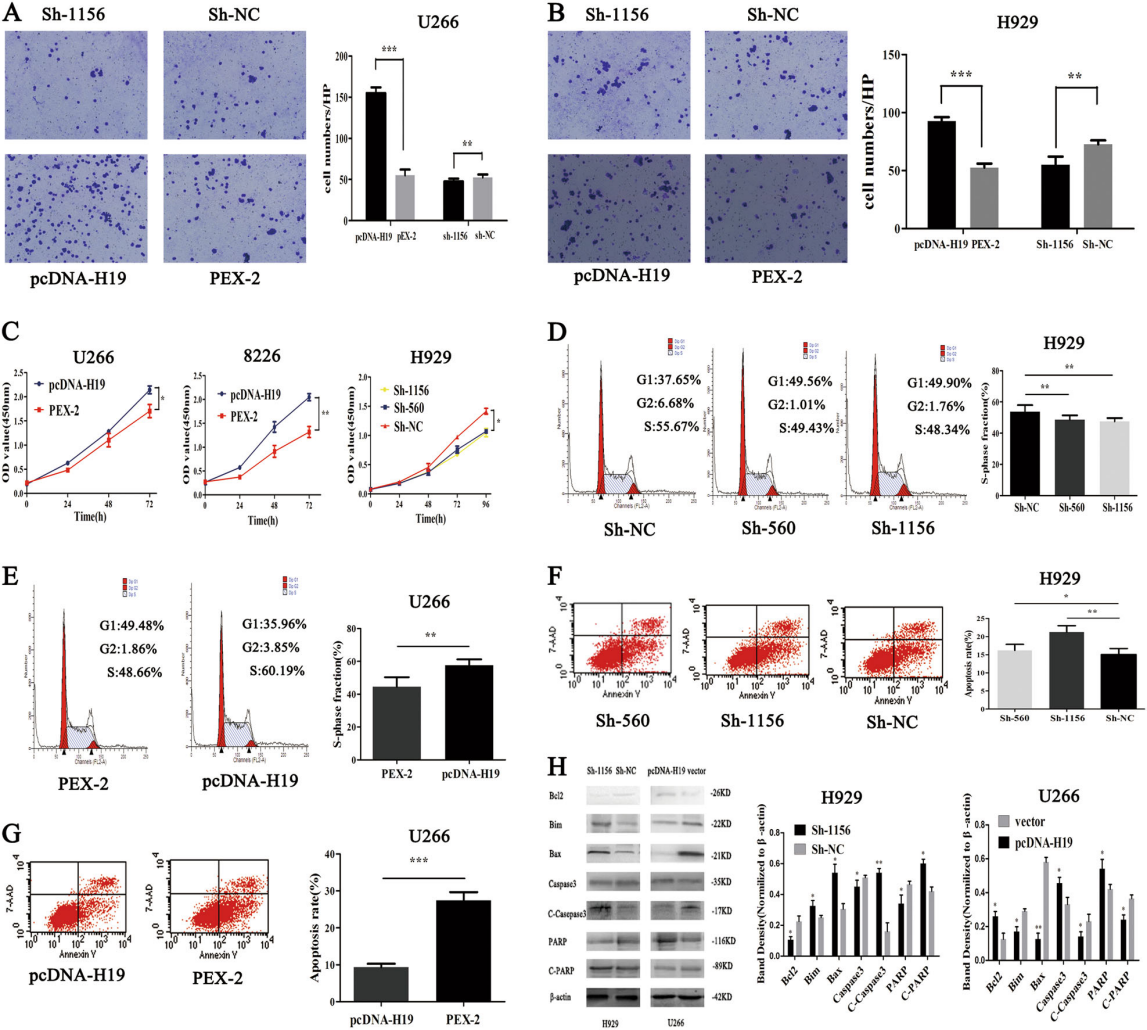
The effect of abnormal expression of H19 on the malignant biological phenotype of MM cells. **a** H19 overexpression promoted migration of U266 cells, and knockdown of H19 inhibited cell migration (*P* < 0.05). **b** H19 overexpression promoted migration of H929 cells, and knockdown of H19 inhibited cell migration (*P* < 0.05). **c** H19 overexpression enhanced cell viability, and H19 knockdown decreased cell viability (*P* < 0.05). **d** S phase arrest of H929 cells after H19 knockdown (*P* < 0.05). **e** H19 overexpression promoted cell cycle progression of U266 cells (*P* < 0.05). **f** H19 knockdown promoted apoptosis of H929 cells (*P* < 0.05). **g** H19 overexpression inhibited apoptosis of U266 cells (*P* < 0.05). **h** Detection of cell related apoptotic proteins by western blot

Knowing that H19 expression was the lowest in U266 and the highest in H929 (Figure S1), U266 was used to do the overexpression study and H929 was used to do the knockdown study in the subsequent experiments by transfecting Sh-560, Sh-1156, Sh-NC, pcDNA-H19, and PEX-2 into H929 and U266 cell lines, respectively. The transfection efficiency was then detected 48 h after transfection (Figure S2). Next, a transwell experiment was performed to investigate the effect of H19 on the cell migration ability. The results with the two cell lines showed that the cell migration ability was decreased and the cell migration ability was increased significantly after knocking down H19 and overexpressing H19, respectively (Fig. 1a, b). The result of subsequent CCK8 experiment showed that cell proliferation was increased in both U266 and 8226 cell lines markedly after overexpressing H19 (*P* < 0.05), and the cell proliferation ability was markedly weakened in H929 cell line after knocking down H19 (*P* < 0.05) (Fig. 1c). Then, changes in cell cycle were detected after transfection by flow cytometry, and the result showed that the proportion of cells in the S phase was decreased significantly compared with that in the normal control group. In addition, the proportion of S cells was increased significantly after overexpression as compared with the control group (Fig. 1d, e). The result of apoptosis detection showed that the early apoptosis rate was significantly increased compared with the Sh-NC group (*P* < 0.05) (Fig. 1f). Compared with the control group, the early apoptosis rate was significantly decreased after overexpression (*P* < 0.05) (Fig. 1g). Seeing that the abnormal expression of H19 was more pronounced in apoptotic cells, changes in apoptosis-related proteins were detected, and the result was the same as expected (Fig. 1h). These results suggested that H19 promoted the malignant biological phenotype of MM cells.

### Prediction and verification of H19 target miRNA

**Fig. 2:**
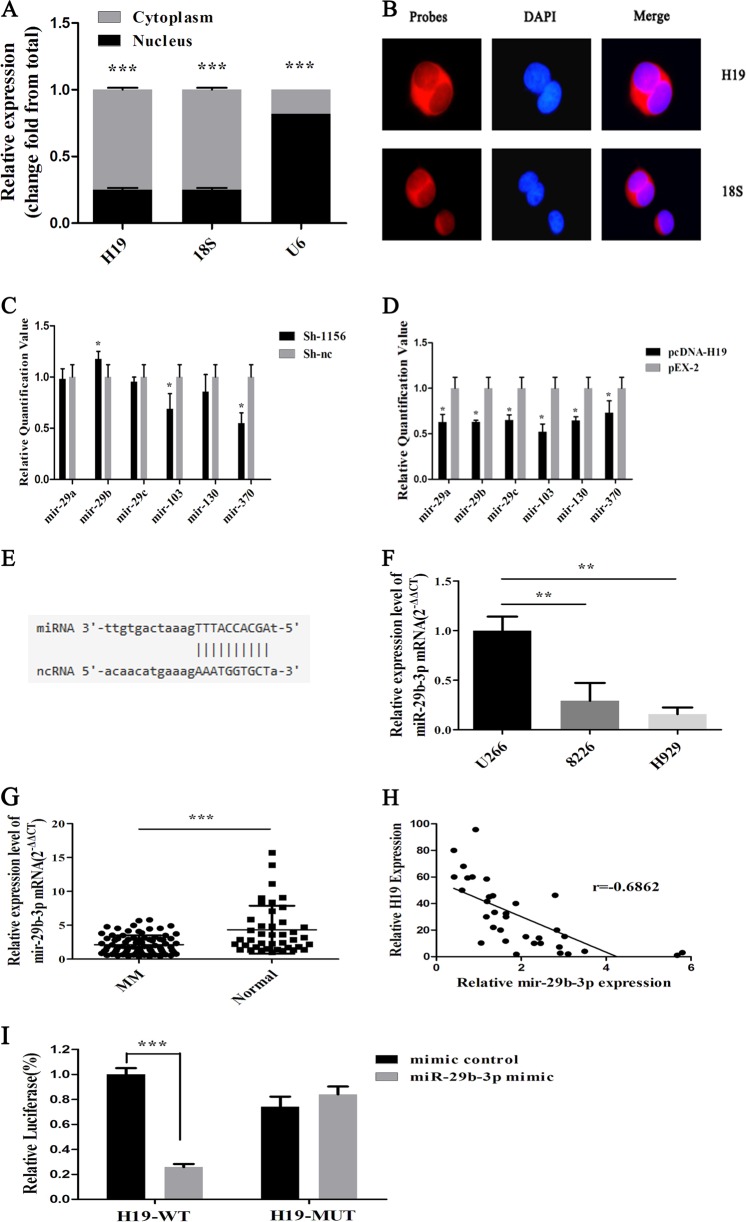
Prediction and verification of H19 target miRNA. **a** The expression level of H19 in H929 cells of caryoplasm. **b** FISH of H19 in H929 cells. **c** Changes in miR-29a, miR-29b, miR-29c, miR-103, miR-130, and miR-370 expression after H19 knockdown. **d** Changes in miR-29a, miR-29b, miR-29c, miR-103, miR-130, and miR-370 expression after H19 overexpression. **e** The binding site of H19 and miR-29b-3p. **f** Expression level of miR-29b-3p in MM cells. **g** The expression level of miR-29b-3p in the serum of MM patients and normal controls. **h** There is a negative correlation between the expression level of mir-29b-3p and H19 in the serum. **i** Detection of the activity of double luciferase reporter gene

To further study the regulatory mechanism of the H19 downstream, we first detected the location of H19 in MM cells by nucleoplasm separation technology and FISH location, and found that most H19 was located in the cytoplasm (Fig. 2a, b). Therefore, we postulated H19 may play a carcinogenic role as a ceRNA. First, we predicted miRNAs that could be combined with H19 by using bioinformatics software StarBase. The result showed that there were six possible miRNAs: miR-29a, miR-29b, miR-29c, miR-103, miR-130, and miR-370. Later, we detected the expression of these six miRNAs by RT-qPCR after overexpression and knockdown of H19 in the cells. The results showed that only miR-29b-3p changed negatively with the change of H19 (Fig. 2c, d). We therefore chose miR-29b-3p for subsequent research (Fig. 2e).

We detected the expression level of miR-29b-3p in MM cells and found that the expression level was contrary to the expression level of H19 in the cells (Fig. 2f). Then, we detected the expression level of mir-29b-3p in serum of 84 MM patients and 42 healthy subjects. The results showed that the expression level of miR-29b-3p in patients was significantly lower than that in healthy controls (*P* *<* 0.0001) (Fig. 2g). To study the correlation between miR-29b-3p and H19, we detected the level of miR-29b-3p and H19 expression in the sera of 34 patients at the same time. The results indicated a negative correlation between miR-29b-3p and H19 (*r* = −0.6862) (Fig. 2h). Based on the above prediction results, we used the luciferase reporter gene to confirm the direct combination of H19 and miR-29b-3p. The result showed that in H19 wild type, the activity of luciferase in miR-29b-3p mimic was significantly lower than that in mimic control (*P* < 0.05), but there was no significant change in luciferase activity in H19 mutant group (Fig. 2i). The results of these experiments clearly indicate that H19 and miR-29b-3p had a negative regulatory relationship.

### H19 played a malignant biological role by inhibiting miR-29b-3p

**Fig. 3:**
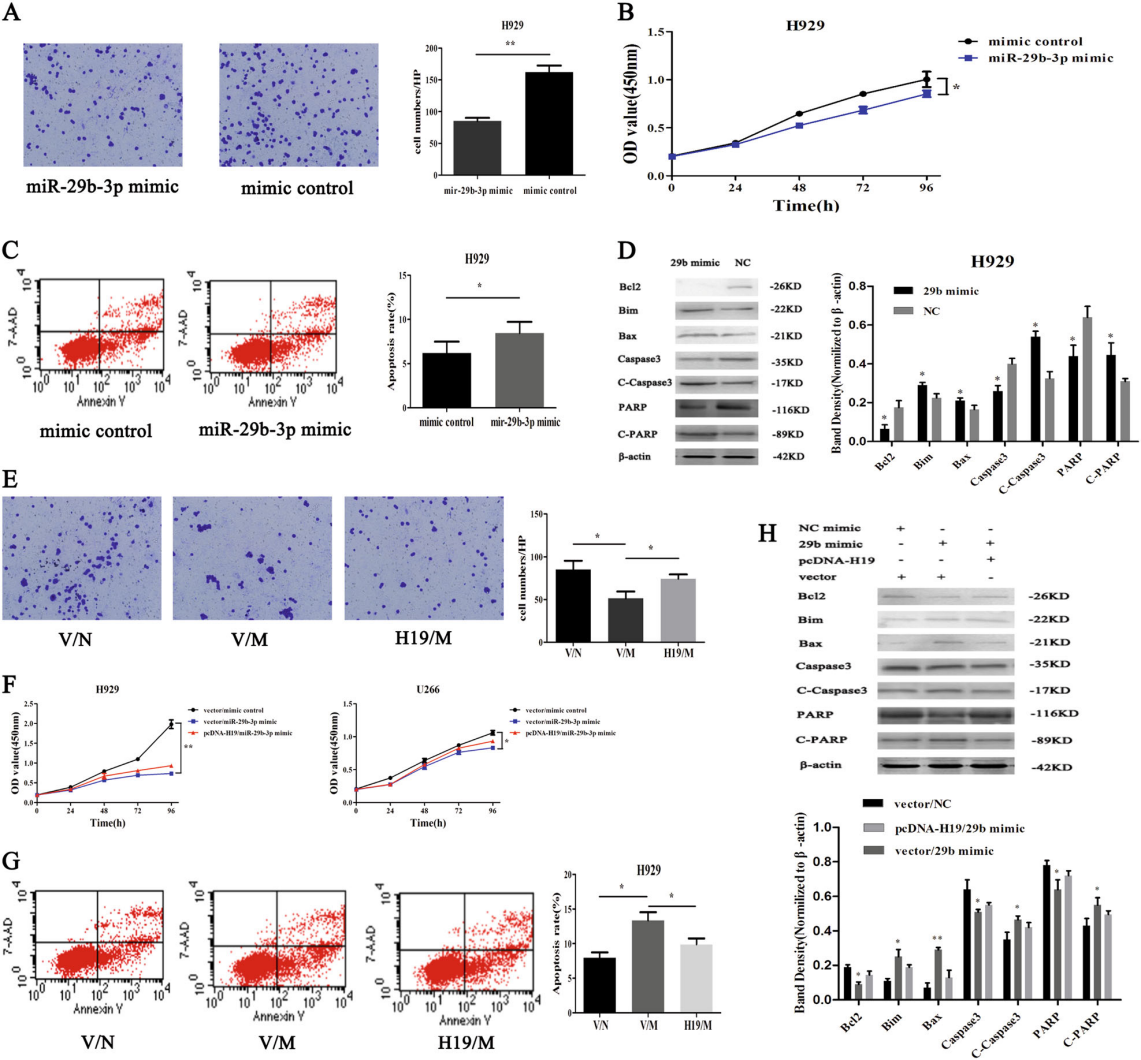
H19 plays a malignant biological role by inhibiting miR-29b-3P. **a** Changes in the number of migrating cells after transfection of miR-29b-3p mimic (*P* < 0.01). **b** Decrease in cell proliferation after miR-29b-3p overexpression (*P* < 0.05). **c** miR-29b-3p overexpression promoted cell apoptosis (*P* < 0.05). **d** Detection of cell related apoptotic proteins by western blot. **e** Change in the number of migrating cells after co-transfection (*P* < 0.05). **f** H19 promoted the proliferation of MM cells through negative regulation of miR-29b-3p. **g** Change in cell apoptosis after co-transfection. **h** Detection of cell related apoptotic proteins by western blot

H19 was reported to regulate tumor metastasis and EMT by miR-29b-3p as competing endogenous RNA in bladder cancer^35^. To verify the effect of mir-29b-3p on the function of MM cells, we carried out the related cell function experiments. As shown in Fig. 2f, the expression of miR-29b-3p was the lowest in H929, and therefore we used H929 to the overexpression experiment by transfecting the mimic control and miR-29b-3p mimic into H929 cell lines and detecting the transfection efficiency after 48-h transfection (Figure S3). Then, we conducted the transwell assay to test the effect of mir-29b-3p on cell migration ability. The results showed that the number of migratory cells in the mimic group was significantly smaller than that in the control group (*P* < 0.05) (Fig. 3a). In addition, the cells were equally inoculated in 96-well plates according to the transfection group. Then the absorbance value was detected at 450 nm at 0, 24, 48, and 72 h. The result showed that cell proliferation in the mimic group was significantly lower than that in the control group (*P* < 0.05) (Fig. 3b). in addition, changes in cell apoptosis were detected in transfected cells by flow cytometry and the results showed that the apoptosis rate in the mimic group was significantly higher than that in the control group (*P* < 0.05) (Fig. 3c). The apoptosis-related proteins are shown in Fig. 3d. All these results indicate that miR-29b-3p acted as a tumor suppressor.

To determine whether H19 promoted the phenotype of MM cells by inhibiting the expression of miR-29b-3p, we co-transfected miR-29b-3p mimic, pcDNA-H19 and their respective controls (mimic control and vector) in H929 cells. The cells were added to the chambers according to the transfected group. The number of migrating cells was observed 24–48 h after co-transfection. The results showed that the number of migrating cells in the vector/miR-29b-3p mimic group was decreased significantly as compared with that in the vector/mimic control group, while the number of migrating cells was increased to a certain extent when pcDNA-H19 was added (Fig. 3e). Similarly, CCK8 results showed that the proliferation ability of cells in the vector/miR-29b-3p mimic group was significantly weakened as compared with that in the vector/mimic control group, and the ability of cell proliferation was restored after addition of pcDNA-H19 (Fig. 3f). The cell apoptosis rate was detected by flow cytometry. Not surprisingly, the results showed that the early apoptosis rate in the vector/miR-29b-3p mimic group was increased significantly as compared with that in the vector/mimic control group, but it was inhibited to some extent when pcDNA-H19 was added (Fig. 3g). In the same way, apoptosis-related proteins underwent changes as expected (Fig. 3h). These results demonstrated that H19 regulated malignant biological phenotypes of MM cells via miR-29b-3p as a competing endogenous RNA.

### The target protein MCL-1 of miR-29b-3p

**Fig. 4:**
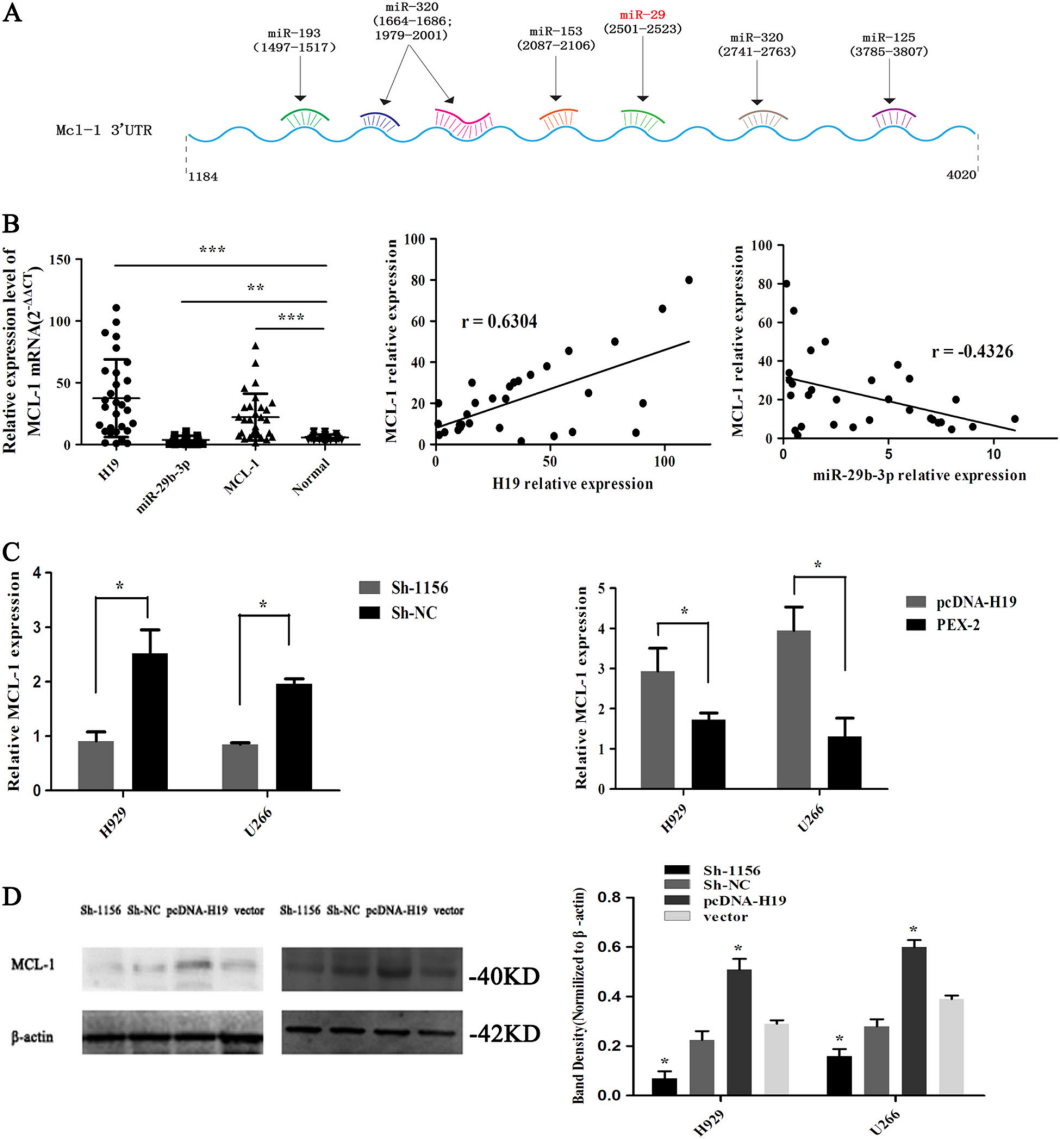
The target protein MCL-1 of miR-29b-3p. **a** The 3′-UTR of MCL-1 has the binding site of miR-29b-3p. **b** Expression of H19/miR-29b-3p/MCL-1 in the serum of BTZ-resistant MM patients. **c** Changes in the mRNA level of MCL-1 by RT-qPCR. **d** Western blot detected the change in the protein level of MCL-1

MCL-1 is a special protein that involved in controlling cell apoptosis. It can help tumor cells escape and survive drug attacks, which is known as chemotherapy resistance. In the present study, we predicted the target protein miR-29b-3p and identified its binding site with MCL-1 by using bioinformatics software (Fig. 4a), confirming the direct regulatory relationship between miR-29b-3p and MCL-1 as reported in the literature^36^. It was found in our previous study that H19 was highly expressed in the sera of BTZ-resistant patients^34^, and we speculated whether MCL-1 was involved in BTZ resistance. In the present study, we focused on the role of the H19/mir-29b-3p/MCL-1 regulation axis in BTZ resistance in MM patients.

To confirm the involvement of H19/miR-29b-3p/MCL-1 regulation axis in MM resistance, we collected the sera from BTZ-resistant patients to detect the expression level of H19, miR-29b-3p and MCL-1. It was found that MCL-1 was highly expressed in the sera of drug-resistant patients and was positively correlated with H19 and negatively correlated with miR-29b-3p (Fig. 4b). Next, we verified the changes in the mRNA and protein levels of MCL-1 in MM cells. First, pcDNA-H19, PEX-2, Sh-1156, and Sh-NC were transfected to H929 and U266 cells to detect the changes of mRNA level in MCL-1. Meanwhile, we collected the protein extracted from the cells after 72-h transfection to detect changes in MCL-1 protein. The results showed that overexpression or knockdown of H19, which resulted in upregulation or downregulation of MCL-1, and the level of MCL-1 protein was consistent with mRNA level (Fig. 4c, d), indicating that H19 could positively regulate the expression of MCL-1.

### H19/miR-29b-3p/MCL-1 regulation axis participated in the regulation of BTZ resistance in MM cells

**Fig. 5:**
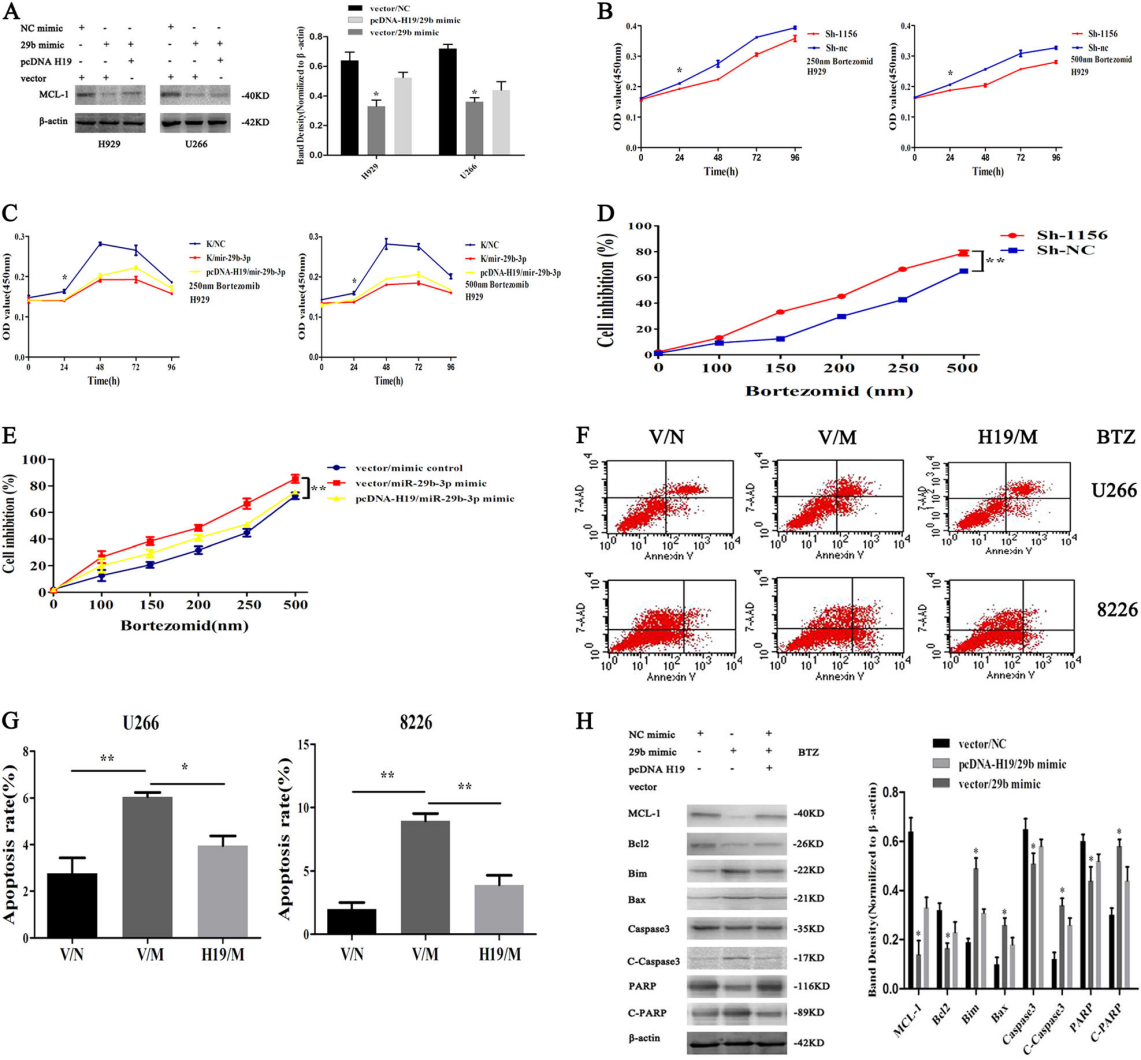
H19/miR-29b-3p/MCL-1 regulation axis participates in the regulation of BTZ resistance in MM cells. **a** Effect of H19/miR-29b-3p co-transfection on MCL-1. **b** After knocking down H19, cell viability was decreased after treatment with 250 nm and 500 nm BTZ. **c** After co-transfection of H19/miR-29b-3p, the sensitivity to BTZ was inhibited to a certain extent after adding pcDNA-H19. **d** The cell inhibition rate was significantly higher with different concentrations of BTZ when the H19 was knocked down compared with the NC group. **e** After co-transfection of H19/mir-29b-3p, the inhibition rate in miR-29b-3p mimic group was higher than that in mimic control group, and the inhibition rate of pcDNA-H19 was decreased to some extent after adding pcDNA-H19. **f** and **g** H19/miR-29b-3p/MCL-1 regulation axis inhibited apoptosis and caused resistance. **h** Changes in MCL-1 and apoptosis-related proteins in MM cells by western blot

To determine whether H19 promoted MCL-1 translation by competing with miR-29b-3p, miR-29b-3p mimic, pcDNA-H19, and their respective controls (mimic control and vector) were co-transfected into H929 cells and U266 cells. After 72 h, cells were collected and the protein level of MCL-1 was detected by western blot. The result showed that the expression of MCL-1 in the miR-29b-3p mimic group was significantly weaker than that in the mimic control group, while the protein level of MCL-1 was enhanced after the addition of pcDNA-H19 (Fig. 5a).

**Fig. 6:**
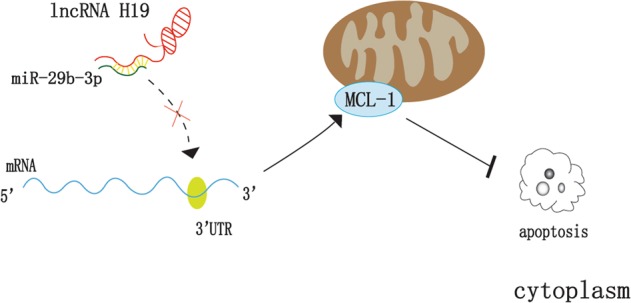
Mechanism of the H19/miR-29b-3p/MCL-1 regulation axis in promoting BTZ resistance in MM cells. The high expression of H19 in MM can be used as a ceRNA to inhibit miR-29b-3p, thus attenuating the inhibitory effect of miR-29b-3p on the target protein MCL-1, which causes a large number of translations of MCL-1 to inhibit apoptosis and induce BTZ resistance

In order to verify whether the H19/miR-29b-3p/MCL-1 regulation axis was involved in BTZ resistance of MM cells, IC50 of BTZ in H929 cells was measured at 250 nm. After knocking down H19, 250 nm and 500 nm BTZ was added. After 24-h processing, cells were seeded into 96-well plates according to the transfected group. After inoculation, the absorbance value was detected at 450 nm at 0, 24, 48, 72, and 96 with microplate Reader. Similarly, after co-transfection of H19/miR-29b-3p, the absorbance values under 450 nm were treated by these methods. The results showed that the absorbance was significantly lower than that in the Sh-NC group at each time point after knocking down H19 (Fig. 5b). Co-transfection of H19/miR-29b-3p showed that the sensitivity to BTZ was inhibited to a certain extent after adding pcDNA-H19 (Fig. 5c). In addition, cells were treated with different concentrations of BTZ to detect the inhibitory effect of BTZ on cell growth. The results showed that compared with the NC group, the cell inhibition rate was increased significantly after knocking down H19 (Fig. 5d). Co-transfection of H19/miR-29b-3p showed that the inhibition rate in the miR-29b-3p mimic group was higher than that in the mimic control group, and the inhibition rate of pcDNA-H19 was decreased to some extent after adding pcDNA-H19 (Fig. 5e). Finally, after 48-h co-transfection, BTZ was added to the cells for 24 h, and then cells were collected and detect for apoptosis by flow cytometry. The results showed that miR-29b-3p overexpression significantly promoted apoptosis of U266 and 8226 cells, and addition of H19 plasmid decreased the percentage of apoptotic cells significantly (Fig. 5f, g). Besides, there was the same change in protein levels (Fig. 5h). Taken together, H19 promoted MCL-1 translation by targeting miR-29b-3p directly in MM cells, thereby inhibiting cell apoptosis and decreasing the sensitivity of cells to BTZ, resulting in acquired drug resistance (Fig. 6).

### LncRNA H19 promoted tumor growth in vivo

**Fig. 7:**
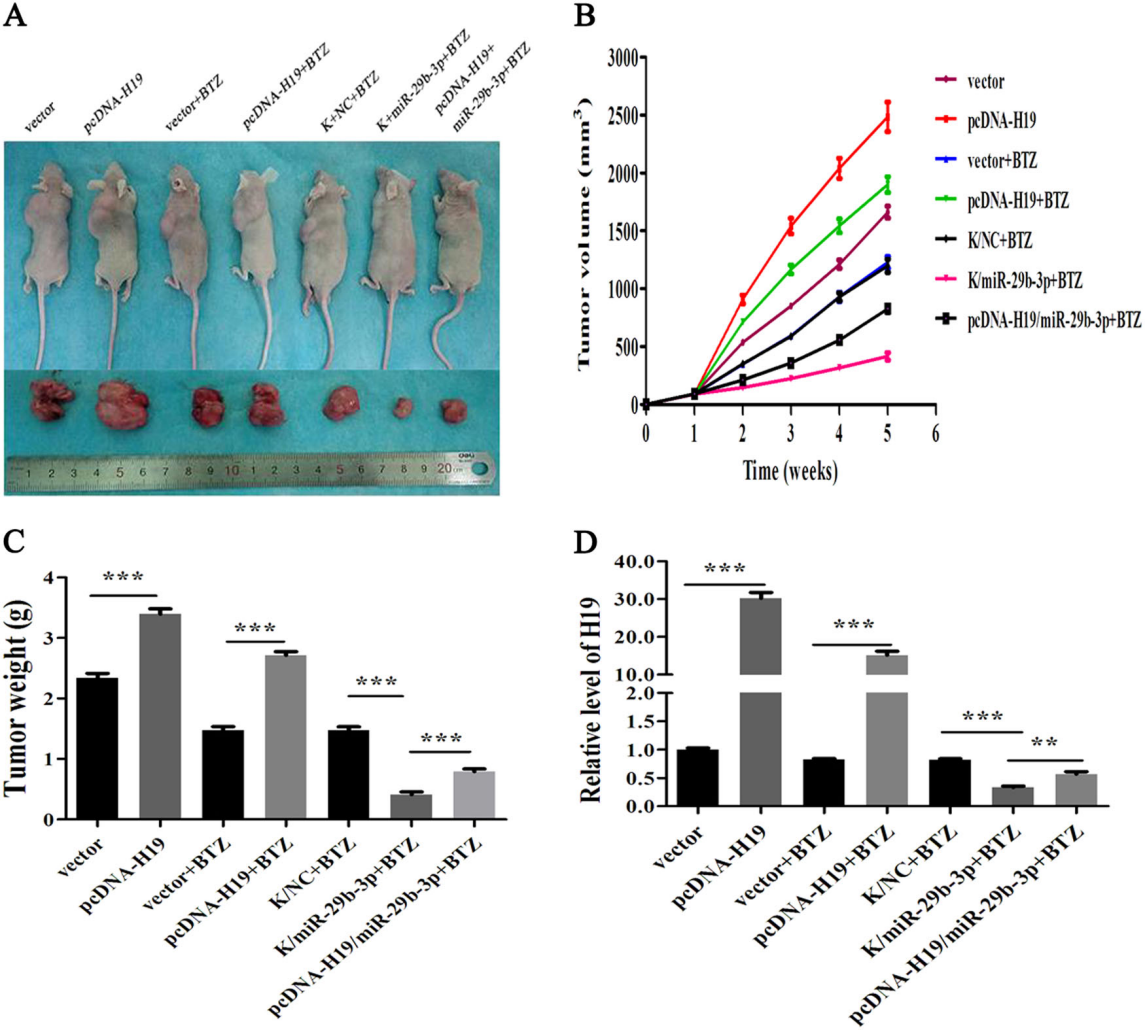
H19 promoted tumor growth and resisted bortezomib to a certain extent in vivo. **a** Photos taken after subcutaneous tumors removal in nude mice. **b** Tumor volumes were measured once a week. **c** The weight of the subcutaneous tumors after removal. **d** H19 expression in tumor tissues were detected by qRT-PCR. (*p* < 0.05)

To investigate the roles of H19 on tumor growth in vivo, we constructed a subcutaneous tumor model. Male athymic BALB/c mice, 5-week-old, were randomly divided into three groups. The first group was overexpression and control group (pcDNA-H19, vector), the second group was bortezomib group (pcDNA-H19 + BTZ, vector + BTZ), and the third group was co-transfection group treated with bortezomid (K/NC + BTZ, K/miRNA-29b-3p + BTZ, pcDNA-H19/microRNA-29b-3p + BTZ). The detailed operation of the experiment was as mentioned above. Results showed that tumor growth rate was significantly faster in the H19 overexpression group than in the control group; bortezomib treatment still showed that the overexpression group was faster than the control group; and in the third group we saw that miRNA-29b did inhibit tumor growth, but when pcDNA-H19 was added, the growth rate of the tumors increased again. The weight and volume of tumors were consistent. (Fig. 7a–c, *p* *<* 0.05). Moreover, after removing the tumors, we detected the expression level of H19 in tumor tissues by qRT-PCR as shown in Fig. 7d. These results indicated that H19 can promote tumor growth and had a certain degree of resistance to bortezomib.

## Discussion

MM is the second common malignant tumor of the hematologic system, characterized by abnormal proliferation of plasma cells in the bone marrow. Most MM patients have anemia, osteolytic lesions, renal damage, hypercalcemia, immune dysfunction, and other clinical manifestations^37^. Almost all currently available treatments for malignant tumors including chemotherapy, targeted therapy, and immunotherapy are confronted with a challenging problem of drug resistance. But as there is little knowledge about the exact mechanism of drug resistance, no molecular target for drug resistance or effective strategy for drug resistance has been put into clinical practice. There are two major categories of drug resistance: primary (endogenous) and acquired (secondary). In clinical practice, primary resistance to certain specific molecular target inhibitors is relatively rare. And acquired resistance is very common, which is a key problem in the current MM targeting therapy.

Current research shows that lncRNAs have four typical molecular functions as signal, decoy, operon, and scaffolding. In the study of MM, lncRNAs have been used as competitive endogenous RNAs or a miRNA sponge. It competes with miRNA response elements to inhibit the function and activity of miRNA, thereby regulating miRNA at the post transcriptional level and regulating its target protein level. Chen et al.^38^ reported that CCAT1 acted as a molecular sponge of miR-181a-5p to promote MM progression by regulating the expression of HOXA1. Meng et al.^21^ found that CRNDE was highly expressed in MM tissue specimens and cells, and was associated with tumor progression and poor prognosis. CRNDE was found to induce proliferation and anti apoptosis through competing with miR-451^21^. All these studies suggested that the ceRNA-based regulatory network played a very important role in the progression of MM.

It was demonstrated in the present study that most H19 was located in the cytoplasm of myeloma cells, suggesting that H19 may regulate the process of drug resistance in MM via ceRNA. We therefore postulated by bioinformatics that miR-29b-3p may be the target miRNA of H19, and MCL-1 may be the target protein of miR-29b-3p. MCL-1 is a protein with a very short half-life and degraded by proteasome through ubiquitination. In addition, it is a member of the anti apoptosis protein Bcl-2 family and highly expressed in many kinds of tumors. The abnormal expression of MCL-1 was reported to be a cause of resistance to multiple chemotherapeutic drugs^39^. MCL-1 has a variety of unique functions and features, making it the most unique member of the antiapoptotic Bcl-2 family. MCL-1 has a very short half-life of less than 1–4 h due to the close regulation by multiple ways to the transcription, translation and degradation of MCL-1. Structurally, the N-terminus of Mcl-1 is also different from other antiapoptotic Bcl-2 proteins. It contains two harmful regions rich in proline, glutamic acid, serine, and threonine. In fact, the N end region can serve as a regulating area of MCL-1 turnover, location, and phosphorylation. Therefore, it can provide a mechanism to rapidly adjust the expression of MCL-1 in response to various environmental and cellular effects. There is ample evidence that MCL-1 is an important tumor target. For example, MCL-1 overexpression was found to be one of the most common genetic variants in human lung and liver cancers^40^. In addition, MCL-1 overexpression induced resistance to Bcl-2 inhibitors and some widely applied anticancer therapies, including paclitaxel, vincristine, and gemcitabine. Studies also demonstrated that silencing Mcl-1 could restore the sensitivity of drug-resistant cells^41,42^. It was highly expressed in MM cells as one of the major survival factors of MM cells and was involved in the process of tumor resistance, but the specific mechanism of its drug resistance is not clear.

In our study, we further analyzed the expression patterns of three molecules H19, miR-29b-3p, and MCL-1 in serum of MM patients with recurrent BTZ resistance. The result of correlation analysis showed that both H19 and MCL-1 were highly expressed in MM patients, and there was a positive correlation between them. In addition, the expression of miR-29b-3p was low in MM patients and negatively correlated with both H19 and MCL-1, suggesting that there may be some connection between the three molecules in the process of MM resistance. Luciferase activity test suggested that miR-29b-3p was a target miRNA of H19 and there was a miR-29b-3p binding site in 3′-UTR of MCL-1^36^. To verify this hypothesis, we used plasmids and ShRNA techniques to explore the effect of H19 in terms of the biological behavior in MM cells. Besides, we used flow cytometry, luciferase reporter vector, western blot and other techniques to explore the role of the H19-miR-29b-3p-MCL-1 regulatory axis in the process of MM BTZ resistance. The results showed that after knocking down H19, the proliferation and migration of myeloma cells were inhibited and cell apoptosis was enhanced markedly, whereas the expression level of miR-29b-3p and H19 in serum was opposite. The tumor suppressor effect of miR-29b-3p was partly reversed by H19 overexpression after co-transfection, and the mRNA level and protein level of MCL-1 were positively correlated with H19. After BTZ treatment, the overexpression of H19 significantly inhibited cell apoptosis and reduced the sensitivity of cells to the drug. Moreover, in vivo experiments, we showed that overexpression of H19 could significantly promote tumor growth and counteract the antitumor effect of miR-29b. Importantly, H19 was resistant to bortezomib to some extent. These data indicated that H19 might function as an oncogene in MM progression.

## Conclusions

In summary, we demonstrated that H19 inhibited apoptosis of MM cells and promoted BTZ resistance by regulating the translation of MCL-1 and targetedly silencing miR-29b-3p, suggesting that H19/miR-29b-3p/MCL-1 may be a novel and promising therapeutic target for coping with drug resistance in MM treatment.

## Electronic supplementary material

Supplements

## Data Availability

Data and materials are available.
