# Supplementary material for: LncRNA H19 overexpression induces bortezomib resistance in multiple myeloma by targeting MCL-1 via miR-29b-3p
Source: Cell Death Dis. 2019 Feb 6;10(2):106. doi: 10.1038/s41419-018-1219-0 (PMC6365572; doi:10.1038/s41419-018-1219-0)
Supplement: Supplementary file 1 — Supplements [file 41419_2018_1219_MOESM1_ESM.pdf]

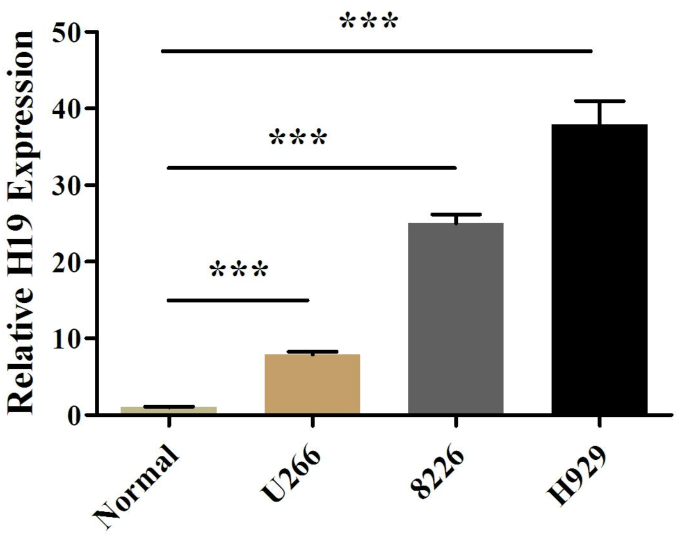

Figure S1. Expression level of H19 in MM cells

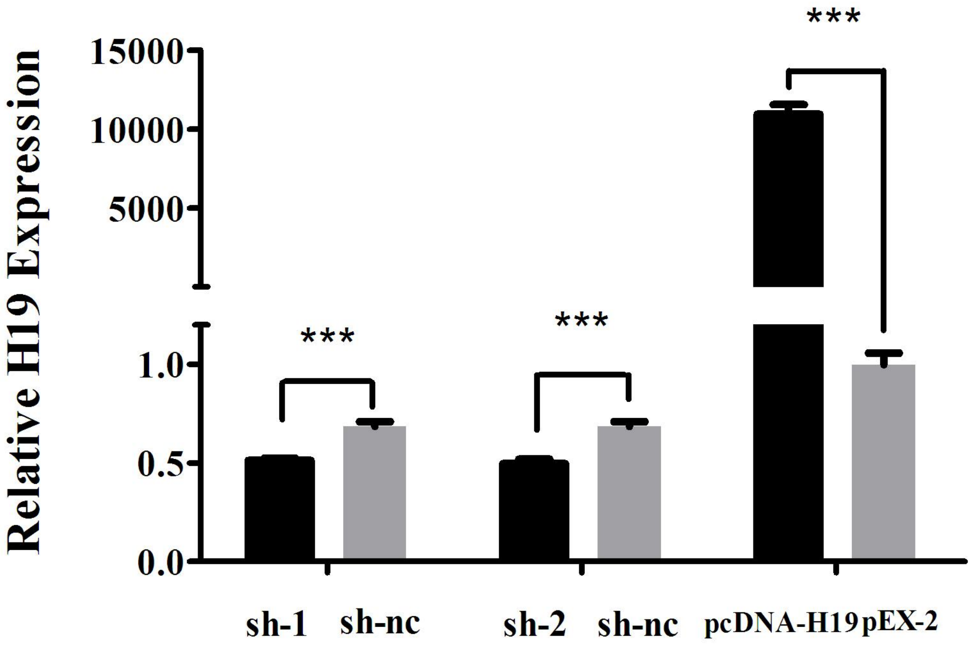

Figure S2 . Transfection efficiency of H19

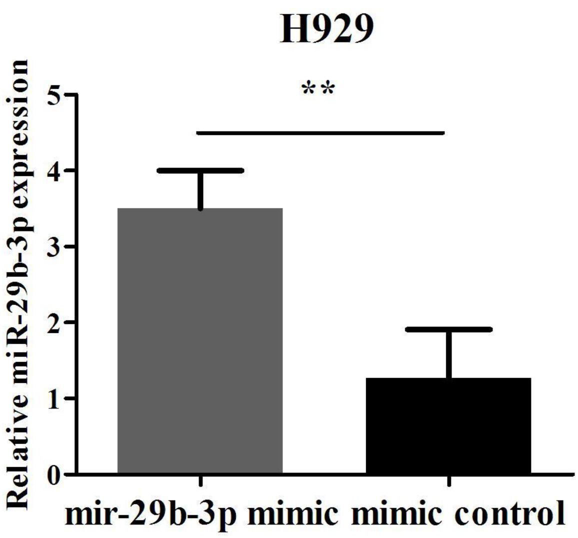

Figure S3. Transfection efficiency of miR-29b-3p
